# Gastrointestinal Infections in Deployed Forces in the Middle East Theater: An Historical 60 Year Perspective

**DOI:** 10.4269/ajtmh.15-0200

**Published:** 2015-11-04

**Authors:** Mark S. Riddle, Stephen J. Savarino, John W. Sanders

**Affiliations:** Naval Medical Research Center, Silver Spring, Maryland

## Abstract

Infectious diarrhea has been among the most common maladies of military deployments throughout time. The U.S. military experienced a significant burden from this disease in the middle eastern and north African campaigns of World War II (WWII). This article compares patterns of disease experienced in WWII with the recent military deployments to the same region for Operation Iraqi Freedom and Operation Enduring Freedom (OIF/OEF). Remarkable similarities in the prevalence and risk factors were noted, which belie the assumed improvements in prevention against these infections. In both campaigns, peaks of diarrhea occurred shortly after arrival of new personnel, which were seasonally associated and were linked to initial lapses in field sanitation and hygiene. It is important to reassess current strategies, especially, in light of emerging evidence of the chronic sequelae of these common infections to include a reemphasis on or reexamination of vaccine development, rapid field diagnostics, treatment algorithms, and antimicrobial prophylaxis.

## Prologue

In 1941, U.S. forces entered World War II (WWII) and joined the allied efforts in the Middle East with the mission to provide logistical support in north Africa and the Persian Corridor. These missions reached peak troop strengths of about 65,000 personnel in 1943. In addition, force totals were augmented by about 25,000 personnel in the Ninth Air Force, which was responsible for missions in Libya, Tunisia, Sicily, Italy, Greece, and Romania. Aside from the geographical region of combat deployment, there are no obvious parallels between the U.S. troop deployment in the Middle East theater during WWII and the recent U.S. deployment to the region in support of Operations Iraqi and Enduring Freedom (OIF/OEF). The recent deployments have consisted of more than twice the troop sizes of the WWII Middle East campaigns and have been characterized not by missions of building roads, railways, and moving supplies, but by defeating counter insurgencies and building up host country institutions. As different as these two major deployments to this region may appear, there is a stark parallel seen with one particular challenge: acute infectious diarrhea.

Although this historical review is limited to the U.S. experience between these two deployments to the same region 60 years apart, it should be clearly stated that the problem of gastrointestinal (GI) infections is not unique to the U.S. soldier and is a problem that has been around for centuries. For the more interested reader, the excellent examinations by Cook,^1^ as well as Connor and Farthing^2^ should be sought out.

## Mortality Transition From Infectious To Trauma Era

With major advances in field sanitation, vaccines, and antimicrobials, the attributable fraction of mortality associated with disease in the age of modern warfare has clearly declined.^3^ Before WWII, infectious diseases were known to cause about four deaths for each one caused by combat. Most notable was the Spanish American War in which 85% of the deaths during the conflict were attributed to typhoid.^4^ During WWII, this ratio dropped to 0.07–1.^5^ It then dropped to 0.02–1 in the conflicts in Korea, Vietnam and in OIF/OEF.^3^ These advances were clearly seen with respect to acute enteric infections (typhoid, diarrhea, and dysentery).^6^,^7^ The authors know of no deaths directly attributable to enteric infections in any military operations in the past 15 years. However, although unlikely to be a primary cause of death in current combat operations, acute enteric infections may indirectly contribute to mortality. This article considers acute GI infections, which is often referred to as travelers' diarrhea or infectious gastroenteritis. A distinction is often made with military diarrhea, which, as with travelers' diarrhea, occurs among individuals traveling from a developed to an underdeveloped area of the world and is caused by the same host of pathogens. What separates military diarrhea from travelers' diarrhea is the population dynamics, the context for treatment, and the impact.^8^ As an editorialist so poignantly phrased it, “[One] cannot fathom the problems attendant from the absolute urgency for relief from explosive vomiting and diarrhea when experienced within an armored vehicle under fire and at ambient temperature of 40°C.”^9^

## Acute Diarrhea in Middle East Theater (WWII)

**Figure 1.**
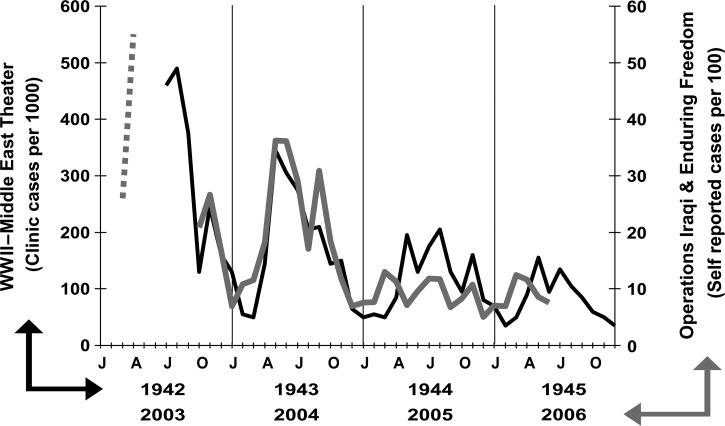
Incidence of diarrhea and dysentery in the Middle East theater during World War II (WWII) (1942–1945) (adapted from Chart 39^5^) and Operations Iraqi and Enduring Freedom (OIF/OEF) (2003–2006) (dashed line adapted from Ref. 17 and solid line from Ref. 10).

For all-cause disease morbidity of WWII, it was said that “[o]pinion concerning progress in the control of communicable diseases is too frequently based on deaths alone, an incomplete measure. Disability and residual defect are other primary considerations….”^5^ Although trailing behind sexually transmitted infections and arthropod-borne diseases, morbidity because of acute diarrhea and dysentery affected many in the Africa–Middle East theater during the war years, accounting for rates of 128 cases per 1,000 person-years and one out of every seven hospital admissions for disease.^5^ A significant risk of disease occurred early on in the war with a diminution over subsequent years with summer peaks in disease incidence (Figure 1

). Laboratory capacity in working up diagnoses of diarrheas and dysenteries was limited, or most often, not attempted. However, the available data suggest that in the Middle East, the incidence of bacillary dysentery was higher than for any other contemporary theater and peaked early in the initial war phase. Specifically, in the Africa–Middle East theater (1942–1945), dysentery (all cause) accounted for 23.3% of all documented acute intestinal infections and shigellosis specifically accounted for about half of these. Furthermore, independent of secular trends, troops first introduced into hyperendemic environments tended to contract bacillary dysentery or unclassified dysentery promptly, a feature also true of the common diarrheas, whereas amebic dysentery was associated with prolonged exposure. However, there may have been a diagnostic bias associated with an emphasis on identification of amebic dysenteries over the period of the conflict.^11^ Furthermore, the inability to microscopically differentiate between pathogenic *Entamoeba histolytica* and the nonpathogenic *E. dispar* may have confounded the attribution of disease, as further evidenced by stool surveys identifying *E. histolytica* in symptom-free individuals returning to the United States.^12^

These high rates of disease occurred despite the comprehensive understanding of basic principles of hygiene, food preparations, and waste disposal. Although adequate knowledge and technology to implement a range of control measures existed at the time, it was a recognized challenge to apply these basic principles throughout a vast military organization dispersed in varied environments where local situations demanded particularized methods. There were several recognized high risk periods and suggestions of future mitigation strategies were proffered, a few of which are worth describing because of their importance and what they portend.^5^ Large epidemics were quite common “[w]ithin a few days or weeks of the first entry of troops (either first arrivals or rotated personnel) into hyperendemic areas, especially under conditions which enabled contact with native populations.” It was thought that early arrivers were not always able to provide ideal sanitation for themselves because of shortages of critical supplies and inadequate or no education in personal protective measures. Moreover, the employment of foreign nationals as food handlers and eating at local establishments were associated with a high incidence of disease. Many of these risk factors were well known, and there was a lack of promulgation or enforcement of strict regulations put in place regarding consumption of high risk foods, such as eating at local establishments and uncooked foods that were washed with “Nile or canal water.”^6^ As one officer recounted, “So long as the linen is of fine quality and clean, and the servants are trained well in the proper serving of the meal, one does not dare to think beyond the kitchen door as to how the cooks prepare the food or as to the condition of the native markets from which they procure the food.”^6^ And as true then as it is today, the attraction to native foods was irresistible to most newcomers and many suffered the consequences. A second tenet of diarrheal disease in war was described from the observations that during periods where troops “were engaged in combat, especially in war of movement” field sanitation and provision of safe food and water activities broke down, and there was a strong correlation between combat intensity and increased incidence of diarrheal disease.^5^ Although it was felt that incidence was lessened when troops in actual combat primarily consumed individual packaged rations, experts concluded that better field sanitation techniques and facilities for frontline troops during actual combat were needed. Other described observations that led to increased incidence of these infections were related to training maneuvers when troops had not yet had sufficient education in field sanitation practices, overcrowding during transportation of troops by rail or over water, and during the construction of fixed bases.

Given the impact of bacillary dysentery, future specific measures for prevention and management of these infections were explored. With the seeming success in typhoid vaccination, vaccines against causes of dysentery were accorded a priority. Early diagnosis was considered important and limited by the technology of the time, so better rapid field diagnostics were needed. Mass chemoprophylaxis appeared to demonstrate encouraging results in some early experiments, but the recognition of acquired resistance to sulfanilamide gave caution to the use and potential misuse of this strategy.

## Acute Diarrhea in the Middle East (2003–Present)

Fast forward 60 years to the next sustained major combat operation in this Middle East region.† †Although the historical impact of acute diarrheal disease (57% attack rate) in the 1990 Persian Gulf War is hardly done justice by a footnote, an emphasis on sustained combat in an austere environment of a similar region is used here for illustrative purposes and the reader is directed to other work for description of acute diarrheas in this earlier operation (Hyams and others 1991). Although tempting, it would be inappropriate to compare directly these operations as there are factors of disease definitions, reporting, and access to care that confound comparisons. That stated, it appears that despite improved sanitation, provision of safe food, and other public health interventions, there has been a minimal reduction in disease incidence in OIF/OEF as compared with WWII, with overall disease and non-battle injury (DNBI) rates of infectious GI visits (the given medical surveillance category for all acute diarrhea, dysentery, and vomiting illness) between March 2003 and June 2006 of approximately 146 cases per 1,000 person-years.^14^ Even if there has been a decline in incidence, there is no question that diarrhea, dysentery, and gastroenteritis were and are still all too frequent an occurrence. In at least one study from a U.S. Army medical facility during OIF in the period after completion of major ground combat operations (October 2003–June 2004), diarrhea (18%) trailed only cellulitis (29%) as cause for hospital admission.^15^ In a study of nearly 5,000 admission records at a British Military Field Hospital in southern Iraq during the first 12 months of military operations, gastroenteritis (31%) was the leading cause of admission.^16^ Even with these dissimilarities between incidence estimates of WWII and OIF/OEF, it would be reasonable to say that the relative burden of infectious GI illness is similar between these deployments.

With this caveat, it is instructive to examine the secular and seasonal trends of acute GI infections between WWII and current operations in the Middle East region. Although monthly DNBI rates of infectious GI medical visits are not publicly available, Figure 1 details rates of self-reported diarrhea obtained from U.S. military personnel throughout the region during in-processing medical screening at the rest and recuperation program in Doha, Qatar.^17^ Despite the distinction in scale and measure between the two combat eras, there are some remarkable similarities and some important differences when looking at the overlaid trends. Similar to WWII, distinct temporal and seasonal patterns of disease are notable with the highest disease incidence occurring early in combat deployments and during the summer months. In OIF/OEF, the incidence of diarrhea during the combat phase was twice that of the pre-combat phase during the initial invasion.^18^ And as history often repeats, the application of preventive interventions during initial phases of deployment proved challenging (Box 1

Box 1 **Similarities in hygiene and field exposure between World War II (WWII) and Operations Iraqi and Enduring Freedom (OIF/OEF)**

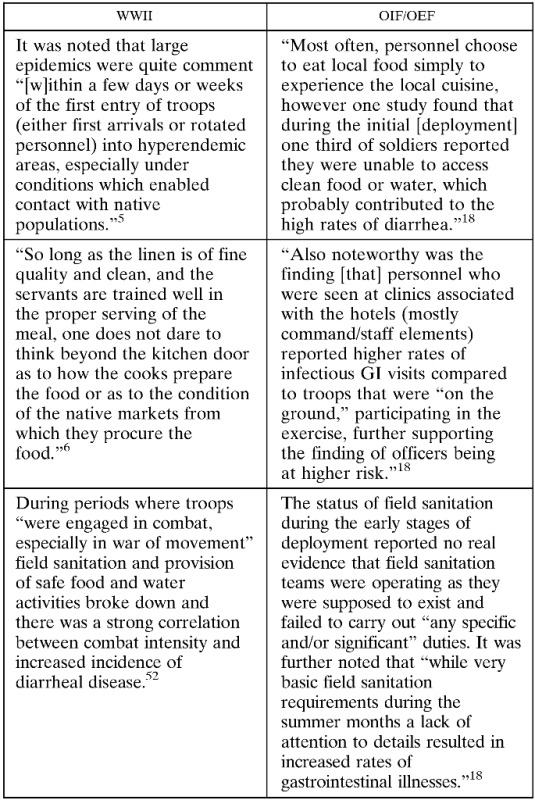

).^19^,^20^ Logistical issues and shortages in environmental health technicians prevented the achievement of some goals, but it was also recognized that soldiers developed diarrhea related to poor hygiene infrastructure and eating food from unauthorized sources.^21^ Most often, personnel choose to eat local food simply to experience the regional cuisine. However, one study found that during the initial “combat phase” in Iraq, one-third of soldiers reported that they were unable to access clean food or water, which probably contributed to the high rates of diarrhea.^18^ Such observations underlie the challenge of field sanitation logistics where effective solutions are still needed. It should be mentioned that not all reports were bad. DNBI surveillance showing high rates of infectious GI illnesses was used to emphasize the need for field sanitation improvements that were associated with a subsequent decrease in diarrheal illness rates.^20^

Our understanding of the pathogens associated with diarrhea has improved considerably in the past 60 years.^22^,^23^ However, when it comes to isolation and identification of these infections in the field, we are still often reliant on technologies similar to those used in WWII. Therefore, the etiologic determination in the field was and is rarely undertaken. Nonetheless, three epidemiologic studies among troops deployed to the region have been conducted, which identified multiple outbreaks of nausea and vomiting and severe diarrhea due to noroviruses and *Shigella* species during early phases of the invasion of Iraq,^24^ with subsequent studies identifying enterotoxigenic *Escherichia coli* and enteroaggregative *E. coli* as primary pathogens isolated from troops deploying to Iraq and Afghanistan.^17^,^25^ It is hypothesized that the outbreaks identified early were probably associated with the deficiencies of hygiene allowing outbreaks of organisms requiring small infectious inoculums and person-to-person spread and that later on the more ubiquitous diarrheagenic *E. coli* were found to prevail. Despite the lack of diagnostics being used at present, the emergence of culture-independent methods provide certain promise to alter the way we diagnose and manage acute diarrhea and gastroenteritis in future conflicts.^26^–^28^ A new era with rapid point-of-care diagnostics could be transformative in providing actionable information on the decision to treat individual patients, the treatment choices, as well as providing early diagnosis for outbreaks that could direct targeted and effective public health response in the field.

Although the antibiotic era was in its infancy during WWII, multiple antibiotics have now been demonstrated to be safe and effective in treating acute diarrhea in travelers (including military).^29^ Interestingly, despite effective treatment trials and several consensus guidelines on management utilizing antimicrobials,^30^–^33^ current management of travelers' diarrhea in the deployed military setting leaves considerable room for improvement. A study early in OIF/OEF found that among the troops who sought care for diarrhea, a medication or treatment was only provided about 50–60% of the time. Loperamide was most often prescribed (37%), followed by antibiotics (27%) and bismuth subsalicylate.^21^ Oral rehydration was the only modality provided in 15% of cases. Corroborating these data, a survey of Army physician assistants reported that for clinical scenarios of moderate diarrhea, loperamide or bismuth subsalicylate was prescribed in 36% of patients, oral rehydration alone was provided in 27% of patients, combination antibiotic/loperamide therapy in 25%, and antibiotic alone in 11%.^34^ The reported use of antibiotics (alone or in combination with loperamide) was higher (18% and 45%, respectively) in patient scenarios of severe diarrhea. In a follow-on survey among a broader spectrum of provider type, similar knowledge, attitudes, and practices were noted.^35^ Although perhaps in the scenario of a mild case of diarrhea in a leisure traveler on vacation in a resort setting, management with fluid rehydration and loperamide may be appropriate, a soldier deployed to an austere tropical or desert climate under heavy body armor and physical activity, with fluid requirements often in excess of 16 L/day,^36^ even mild diarrhea should be managed aggressively to prevent unwanted morbidity associated with acute illness and dehydration.

## It Takes “Good Guts” to be a Soldier

Despite the frequent occurrence, it is easy to dismiss these inconvenient GI infections by soldier, provider, and commander when more serious concerns for life and limb, posttraumatic stress conditions, and significant wound infections are real and ever present. Diarrhea and dysentery do not kill, maim, or disfigure. And they are unlikely to lose battles or wars anymore.^37^ However, lost duty days, decreased performance, and health-care utilization are probably underappreciated.^38^ Only about one in five cases of acute diarrhea are captured by current medical surveillance systems,^18^,^21^,^39^,^40^ leaving a large burden of disease that occurs “under the surveillance radar.”

Furthermore, our recognition of the potential sequelae of these common infections is rapidly advancing. Interestingly, probably one of the earliest accounts of post-infectious functional GI disorder was described by Sir Arthur Hurst in his 1918 second edition of *Medical Diseases of the War* where the following is excerpted from his description of colitis and irritability of the colon following dysentery.^41^

“Patients who have recovered from an acute attack of dysentery frequently remain unfit for a considerable period, which may even extend to years. The symptoms are due to the chronic colitis, which may follow either amoebic or bacillary dysentery after the specific infection has died out…In most cases the patient suffers from alternating attacks of constipation and diarrhoea, the latter often being brought on by aperients taken for the relief of the former, or it may follow an indiscretion in diet or exposure to cold…The diarrhoea may only last for a few hours, or it may continue for two or three days, the attacks being separated by intervals of several weeks or months…Sometimes the attacks of diarrhoea cease to occur, but intractable constipation remains and the general symptoms persist, though in a lessened degree.”

Subsequently, similar reports were identified among British soldiers returning from WWII, where multiple endoscopy and fecal examinations revealed an absence of inflammation or infection.^42^,^43^ Systematic reviews of more recent studies have concluded that roughly one out of 10 people who develop travelers' diarrhea will go on to report a post-infectious functional bowel disorder.^44^–^46^ Other less frequent complications such as Guillain–Barré syndrome, reactive arthritis, and inflammatory bowel disease have been described.^47^

With our rapidly expanding knowledge and understanding of genetics and the role of the microbiome in susceptibility to both infections and development of post-infectious sequelae,^48^–^53^ the Civil War era refrain that it takes “good guts” to be a soldier may yet ring true again,^54^ and hopefully with increased understanding we may be able to identify and prevent both the acute and chronic disease morbidity attributed to these infections.^55^

## Conclusion

In the last 60 years, we have made significant strides in our understanding of infectious diarrhea, which makes the striking parallel in the experience of WWII with the recent military deployments even more remarkable. From the WWII experience there was still room for disease prevention optimization through training, regulation, and adequate advance planning. It was astutely concluded that “[f]rom the history of the diarrheas and dysenteries in the United States Army in World War II, many lessons may be learned which will be of value in minimizing future incidence. Doubtless better methods await needed research and development for use when large numbers of troops enter such specialized environments if future unnecessary diarrheal disease in the Army is to be prevented.”^5^ It is evident from a comparison of then and now that this statement largely still holds true and that we must reexamine our current thinking to fully meet the goals of minimizing this burden of disease in future campaigns. Development of strategies, policy, and research direction needs to be informed by a new paradigm in the way we think about this deployment health threat (and threat among the general traveler), which includes both the direct costs of illness, the secondary costs and risks of diminished capabilities, and the potential for long-term costs of sequelae. This should include a reemphasis on or reexamination of the development of vaccines, rapid field diagnostics, treatment algorithms, antimicrobial prophylaxis, and enhancement of our microbiome defenses. We should not stand resigned to the inexorableness of acute enteric disease among deploying troops—our awareness, capability, and mandate to protect the health of those who serve do not allow this.
